# Metformin-Induced Intense Bowel Uptake Observed on Restaging FDG PET/CT Study in a Patient with Gastric Lymphoma

**DOI:** 10.4274/MIRT.020573

**Published:** 2011-12-01

**Authors:** Sabire Yılmaz, Meftune Özhan, Sait Sager, Duygu Yörük Atik, Metin Halac, Kerim Sönmezoğlu

**Affiliations:** 1 Department of Nuclear Medicine, Cerrahpasa Medical Faculty, University of Istanbul, Istanbul, Turkey; 2 Department of Nuclear Medicine, Fatih Sultan Mehmet Hospital, Istanbul, Turkey

**Keywords:** Fluorodeoxyglucose F18, metformin, Positron-emission tomography

## Abstract

A 53-year-old man with a diagnosis of gastric non-Hodgkin lymphoma (NHL) underwent PET/CT scans both prior to starting chemotherapy and immediately following completion of chemotherapy to evaluate the response to therapy. Pre-therapy PET/CT images showed intense FDG uptake in the antral region of the stomach. Biodistribution of FDG was otherwise unremarkable. The patient was started on metformin in the middle of his therapy period to provide glycemic control. Post-therapy PET/CT study performed after 6 courses of chemotherapy showed complete resolution of the disease with no evidence of residual FDG uptake. However, intense and diffuse FDG accumulation is observed in the bowel, which was interpreted as physiological and most probably due to metformin administration. It should be borne in mind that there are a number of physiological variants of FDG biodistribution seen on PET/CT imaging. Recognizing physiologic bowel activity is crucial for the accuracy of PET image interpretation.

**Conflict of interest:**None declared.

## INTRODUCTION

F-18 fluorodeoxyglucose (FDG) positron emission tomography (PET) imaging is a commonly used method for diagnosis or staging of malignancy as well as for evaluating treatment response. FDG competes with glucose for hexokinase phosphorylation to FDG-6- phosphate and FDG-6-phosphate becomes trapped in tissue in proportion to the rate of glycolysis or glucose utilization of that tissue (1). There are a number of physiologic variants including normal physiologic uptake in the head and neck, heart, breast, thymus, liver, spleen, gastrointestinal tract, genital system, urinary collecting system, bone marrow, muscles, and brown adipose tissue. Benign lesions with increased FDG uptake also can be misinterpreted as malignancies (2). In this case, we showed diffuse bowel uptake detected on post-therapy PET/CT in a case of gastric NHL patient who was started on metformin after initial PET/CT.

## CASE REPORT

A 53-year-old man with a diagnosis of gastric non- Hodgkin lymphoma (NHL) underwent PET/CT scans both prior to starting chemotherapy and immediately following completion of chemotherapy to evaluate response to therapy. Pre-therapy PET/CT images showed intense FDG uptake in the antral region of the stomach. Biodistribution of FDG was otherwise unremarkable. The patient was started on metformin in the middle of his therapy period to provide glycemic control. Post-therapy PET/CT study performed after 6 courses of chemotherapy

**Figure 1:**
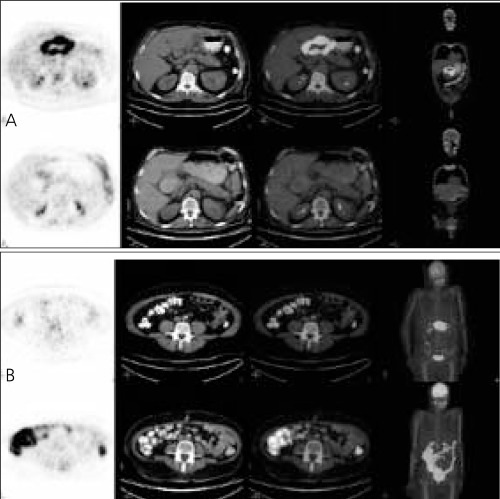
Selected images of pre-therapy and almost identical slices of posttherapyPET/CT seen in upper panel (1a); selected images of pre-therapyand corresponding slices of post-therapy PET/CT in the lower panel (1b)

After a ten-hour fasting (serum glucose level 66 mg/dL), the patient underwent PET/CT imaging from the skull base to mid thigh at 1 hour post injection of 16,6 mCi (614 MBq) FDG using a dedicated full-ring LSO based PET scanner integrated with a 6-slice CT. No intravenous contrast was given for CT portion of the study. However, a 50 mL of iodinated contrast diluted into 1500 ml water was orally administered starting from the midnight, to make the bowel visible on CT scans. The pre-therapy PET/CT images (Figure 1a upper panel) confirmed gastric involvement of the disease with no other pathological FDG accumulation throughout the body including the bowel. On the post-therapy PET/CT images (Figure 1a lower panel), the gastric uptake was completely disappeared compatible with metabolic response to therapy. However, a diffusely increased FDG accumulation was seen in the bowel this time, most probably due to metformin induced changes.

## LITERATURE REVIEW AND DISCUSSION

Dimethylbiguanide metformin or "metformin", a biguanide molecule with a half-life of 6 h, is an orally administered drug that lowers the blood glucose concentration and improves the insulin sensitivity in type 2 diabetes. It is the first-line drug of choice for the treatment of type 2 diabetes. It acts by increasing glucose uptake, oxidation and glycogenesis by muscle, increasing glucose metabolism to lactate by the intestine, reducing hepatic gluconeogenesis and possibly a reducing rate of intestinal glucose absorption (3). An intense diffuse F-18 FDG uptake along the bowel is typical for metformin- induced bowel uptake. Metformin causes significant amount of increased FDG uptake in colon and to a lesser extent in small intestines. Metformin exerts its intestinal effect mainly on mucosa (4). The molecular mechanisms of metformin action are not well understood. However, animal studies indicate that metformin enhances glucose transfer from the vascular compartment into cells of the intestinal mucosa and increases lactate production responsible for enlarge glucose utilization (5). Another study showed that metformin induces GLUT 2 recruitment to the luminal surface by activation of AMPK (AMP- activated protein kinase), resulting in increased glucose uptake by the intestine (6). The other causes of 18F-FDG uptake in the digestive tract are active smooth muscle, metabolically active mucosa, swallowed secretions, or colonic microbial uptake. Uptake in the cecum and right colon is usually higher than the other colonic segments because of the abundance of the lymphocyte cells, which are very glucose avid. There are also other causes of F-18 FDG uptake such as infectious and inflammatory conditions (enterocolitis, diverticulitis), hyperplastic colonic polyps and anorectal hemorrhoids without signs of infection (7). Increased FDG uptake is frequently seen in gastrointestinal system that can sometimes pose interpretative problems. Activity in both large and small bowel is especially problematic as it may obscure malignant lesions. It is important to understand the variable physiologic FDG uptake in the GI tract to improve image interpretation.
